# Microgravity and cancer cells: from molecular mechanisms to therapeutic strategies

**DOI:** 10.1038/s41420-025-02836-8

**Published:** 2026-01-09

**Authors:** Jian Yuan, Sisi Zhang, Yi Xu, Cunman He, Zuchao Cai, Zhiyong Wang, Qianming Chen

**Affiliations:** 1 Stomatology Hospital, School of Stomatology, Zhejiang University School of Medicine, Zhejiang Provincial Clinical Research Center for Oral Diseases, Key Laboratory of Oral Biomedical Research of Zhejiang Province, Cancer Center of Zhejiang University, Engineering Research Center of Oral Biomaterials and Devices of Zhejiang Province, Hangzhou, Zhejiang China; 2 College of Life Science, Zhejiang University, Hangzhou, Zhejiang China

**Keywords:** Cancer microenvironment, Mechanisms of disease

## Abstract

All living organisms on Earth have evolved mechanisms to counteract the effects of gravity. In space, however, shear forces, buoyancy-driven convection, and hydrostatic pressure are either eliminated or significantly reduced. Microgravity disrupts the balance between intracellular structures and external forces, leading to changes at both the cellular and subcellular levels. The absence of gravitational forces in microgravity significantly impacts cellular behavior, including changes in tumor cell morphology, cytoskeletal structure, and gene expression. Research has demonstrated that microgravity induces the three-dimensional aggregation of cancer cells into multicellular spheroids, which more closely resemble in vivo tumors. These spheroids exhibit altered behaviors, including increased apoptosis, autophagy, and reduced proliferation and migration. Such changes suggest that microgravity may offer a promising novel therapeutic approach for cancer treatment. However, the precise underlying mechanisms remain largely unexplored. This review examines current microgravity research platforms and explores how microgravity affects tumor cell molecular and biological behaviors, offering valuable insights into the potential for innovative cancer therapies.

## Facts

- Microgravity profoundly reshapes the physical and biological behaviors of cancer cells, influencing their morphology, cytoskeletal organization, and gene expression.
- The loss of gravitational forces promotes the formation of multicellular tumor spheroids that closely recapitulate the three-dimensional architecture of in vivo tumors.
- Microgravity-induced alterations enhance apoptosis and autophagy while suppressing cell proliferation and migration.
- Simulated microgravity platforms offer robust models for investigating tumor biology and identifying novel therapeutic targets.
- Deciphering the molecular mechanisms underlying these effects may pave the way for the development of gravity-based anticancer strategies.

## Introduction

**Fig. 1:**
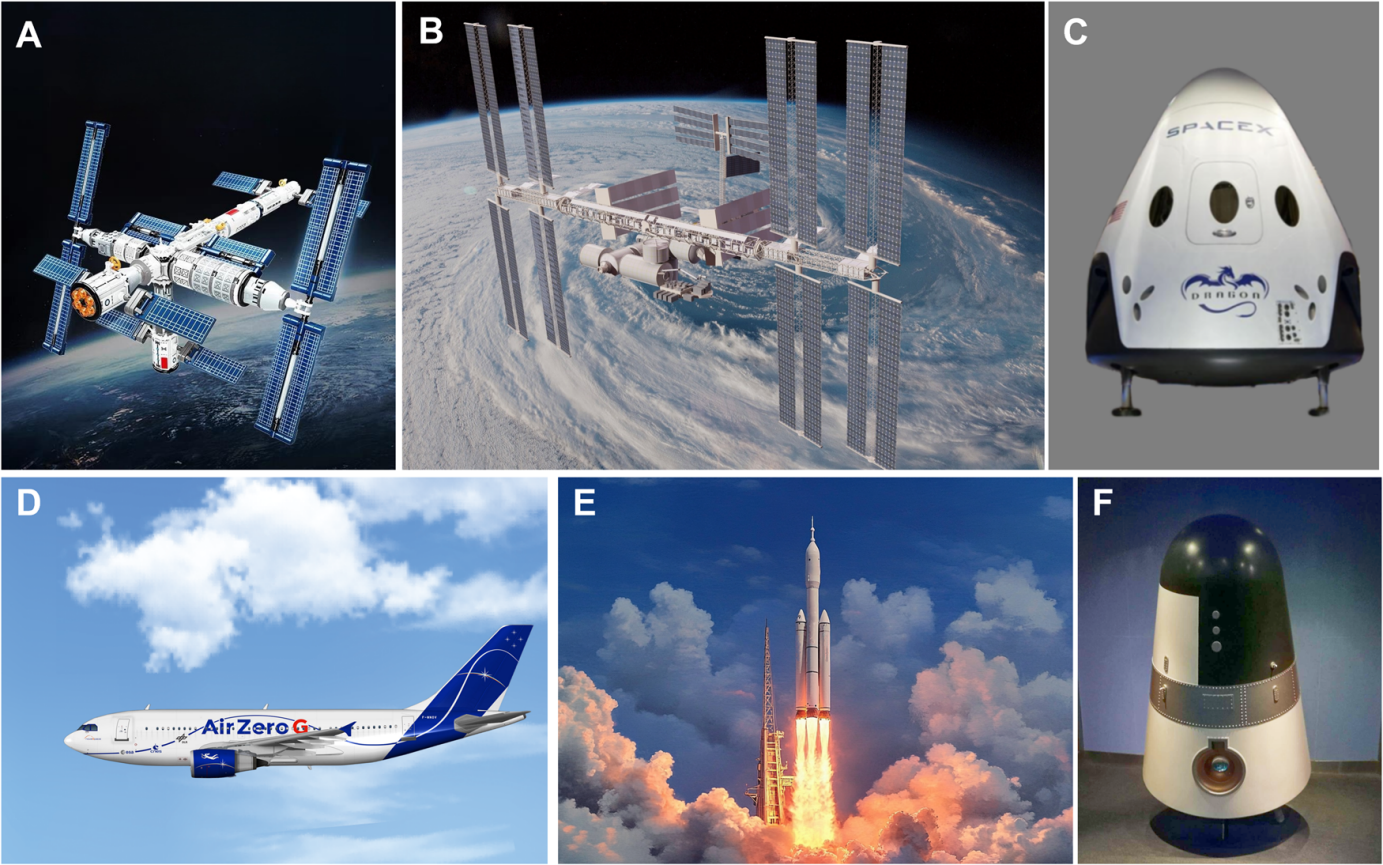
Microgravity research platforms and space facilities. **A** Model of the Tiangong space station laboratory. **B** Model of the International Space Station. **C** Model of the SpaceX Dragon spacecraft. **D** Model of the zero-gravity laboratory aboard the Airbus A300 ZERO-G aircraft by Novespace. **E** Launch site of Tianhe, the core module of China’s space station. **F** Model of the Shijian-10 recoverable satellite.

Microgravity, a unique condition encountered in environments such as parabolic flights, orbiting spacecraft, and space station laboratories, has been a subject of increasing research since the early days of space exploration in the 1960s [1] (Fig. 1). Transitioning from Earth’s gravitational forces to a microgravity environment triggers significant physiological and cellular adaptations, affecting various bodily systems. Documented effects on astronauts in microgravity include osteoporosis, muscle atrophy, cardiac atrophy, increased intracranial pressure, immune system suppression, and cellular reorganization [2–9]. These effects emphasize the importance of studying microgravity’s impact on health at the organ, tissue, cellular, and subcellular levels [10]. Typically, microgravity is considered detrimental to the human body, with various negative effects documented. However, this raises an intriguing question: Could microgravity environments also possess beneficial effects on the body?

Recent research has expanded into exploring the effects of microgravity on cancer cells, revealing that the absence of gravity can influence tumor biology in profound ways. Cancer cells exposed to microgravity environments form three-dimensional multicellular spheroids, a model that more accurately mimics the structure of in vivo tumors compared to traditional two-dimensional cultures [11–14]. These spheroids exhibit altered biological behaviors, such as enhanced apoptosis, autophagy, and suppressed proliferation and migration, suggesting that microgravity may not only provide new insights into cancer progression but also hold potential as a novel therapeutic strategy [11–13, 15].

Cancer cells can proliferate uncontrollably and metastasize to distant organs, leading to severe impacts on human health due to their high morbidity and mortality rates [16–18]. As a leading cause of death, cancer underscores the urgent need for innovative treatment strategies. Microgravity, by influencing cellular and molecular processes, offers a new frontier in cancer research [19]. This review explores current microgravity research platforms, the molecular mechanisms driving cancer cell behavior in microgravity, and the potential of microgravity-based therapies to improve treatment outcomes.

## Microgravity research platforms and equipment

Microgravity environments are typically classified as real or simulated [20]. Real microgravity refers to the sustained weightlessness experienced aboard space stations, while short-term microgravity is achieved during space flights, satellite missions, and spacecraft operations. However, challenges such as radiation interference and high operational costs limit the ability to conduct extensive microgravity research in these environments. To overcome these limitations, researchers have developed ground-based devices that simulate microgravity, aiming to replicate the physiological responses observed in space [21].

**Fig. 2:**
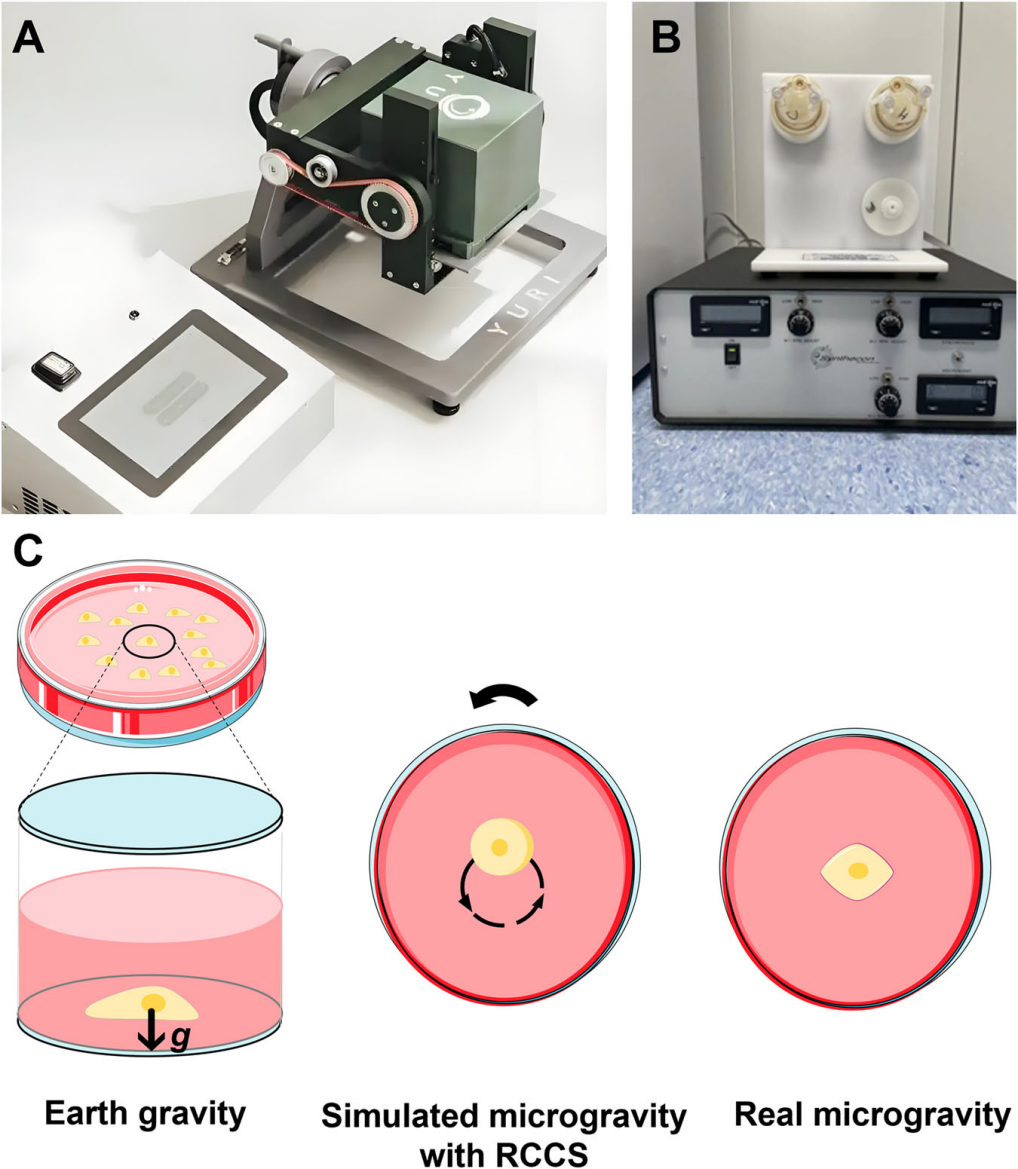
Ground-based microgravity simulation techniques. **A** Random Positioning Machine (RPM), which averages gravitational vectors to simulate microgravity. **B** Rotating Wall Vessel (RWV), including the Rotating Cell Culture System (RCCS), where continuous rotation counteracts gravitational directionality to simulate a microgravity environment. **C** Diagram illustrating the comparison between normal gravity (left), simulated microgravity (center), and real microgravity (right), showing the effects of gravity on cellular behavior in different environments.

Simulated microgravity is commonly achieved through methods such as animal hindlimb unloading, human head-down tilt bed rest, and three-dimensional cell rotation systems [22]. The hindlimb unloading method is widely acknowledged as an effective animal model for simulating microgravity [23, 24], enabling the replication of spaceflight-induced weightlessness effects on various physiological systems [23, 25–27]. For cellular studies, two primary devices are used: the Random Positioning Machine (RPM) (Fig. 2A) [28], and the Rotating Wall Vessel (RWV), including NASA’s Rotating Cell Culture System (RCCS) (Fig. 2B) [29]. The RPM simulates microgravity by averaging the gravity vector to near zero through random movement [30], while the RWV and RCCS simulate microgravity by continuously rotating the vessel, neutralizing directional gravity forces to partially replicate the conditions of a space station (Fig. 2C) [31].

## Effects of microgravity on cellular cytoskeleton and morphology

### Effects of microgravity on cytoskeleton and morphology of cancer cells

Cells are highly responsive to variations in gravity, particularly through changes in their cytoskeletal architecture, which governs shape, adhesion, and mechanotransduction [32–34]. The cytoskeleton, composed of actin filaments, microtubules, and intermediate filaments, plays a fundamental role in maintaining cellular integrity, mobility, and intracellular signaling. Given its involvement in tumor proliferation, progression, and metastasis, understanding how microgravity alters cytoskeletal dynamics is crucial for both space biology and cancer research [32, 33].

Both simulated [35–37] and real microgravity [38–41] induce cytoskeletal reorganization, altering cellular morphology and function. These changes often involve disassembly and redistribution of cytoskeletal components, affecting mechanical stability and intracellular signaling. For example, cytoskeletal alterations in Ewing’s sarcoma cells persist for at least 24 h after exposure to simulated microgravity, demonstrating the long-term impact of gravitational changes on cell structure [42]. Similarly, human breast, lung, and thyroid cancer cells tend to form three-dimensional aggregates or multicellular spheroids under microgravity conditions, which is linked to cytoskeletal rearrangements and gene expression changes [43–46].

A key structural transition involves actin filaments shifting from a longitudinal to a spherical arrangement in human lung and breast cancer cells [45, 46]. This phenomenon aligns with Ingber’s tensegrity model, which suggests that cellular architecture is maintained through a balance of tensile and compressive forces in the cytoskeleton [41, 45]. Disruptions in this balance can lead to altered adhesion, signaling pathways, and tumor progression [41, 45]. Data from both benign and malignant tumor types exposed to short-term microgravity further support this theory, demonstrating that microgravity-induced cytoskeletal disruption may play a role in tumor progression [46–49].

Tumor cells undergo significant morphological remodeling in microgravity, primarily through disruptions in adhesion followed by reorganization into spheroids [50, 51]. This restructuring correlates with actin filament accumulation, which shifts from an elongated alignment under normal gravity to a compact, spherical arrangement under microgravity [43, 50]. Disruptions in microfilaments and microtubules affect key cellular processes, including proliferation, DNA repair, and invasion [37, 52, 53]. Notably, actin filaments play an essential role in mitotic spindle formation, and their rearrangement under microgravity can impair cell division and decrease proliferation rates [54–56].

Distinct cancer cell lines exhibit unique cytoskeletal responses to microgravity. MCF-7 breast cancer cells display cytoskeletal alterations under simulated microgravity, suggesting significant reorganization of actin and microtubules [43]. FTC-133 thyroid cancer cells develop filamentous and lamellar structures in both real and simulated microgravity, indicating structural adaptations to the microenvironment [46]. A431 epidermoid carcinoma cells show a rapid increase in microfilament levels within just 7 min of exposure to real microgravity, highlighting the immediate cytoskeletal response [57].

These findings underscore the cytoskeleton’s role in tumor adaptation under microgravity and suggest that targeting cytoskeletal remodeling could be a promising avenue for future cancer therapies [58]. Given that microgravity conditions mimic aspects of tumor metastasis, understanding how cytoskeletal disruptions affect tumor behavior may inform novel therapeutic strategies, particularly for drug-resistant cancers.

### Effects of microgravity on cytoskeleton and morphology of immune cells

Microgravity conditions have been shown to significantly impact the immune system, potentially altering the responsiveness of immune cells to immunotherapies. The immune system is compromised under microgravity, as evidenced by various studies. For instance, microgravity has been found to impair DNA damage repair in human hematopoietic stem/progenitor cells, which are crucial for generating immune cells necessary for an effective antitumor response [59]. Furthermore, microgravity has been observed to disrupt the signaling pathways in immune cells, such as the NFκB signaling in dendritic cells, which are vital for initiating immune responses [60]. This disruption leads to a less mature dendritic cell phenotype and impaired ability to induce T cell activation, which could hinder the effectiveness of immunotherapies that depend on dendritic cell function [60]. The impact of microgravity on T cells, which are central to many immunotherapies, is also notable. Studies have shown that microgravity can suppress T cell activation and proliferation, which are critical processes for the success of immunotherapies targeting cancer and infectious diseases [61, 62]. This suppression is linked to alterations in cytokine production and signaling pathways within T cells [61, 62]. Moreover, macrophages exhibit altered cytokine secretion and impaired ability to present antigens under microgravity conditions, which could affect the outcome of immunotherapies [63, 64]. Additionally, microgravity-induced alterations in immune cell metabolism and bioactive lipid signaling have been documented. These changes can influence the immune system’s overall homeostasis and responsiveness to therapeutic interventions, including immunotherapies [65]. Lastly, the combined effects of microgravity and other space-related stressors, such as radiation, further complicate immune cell function. This combination can lead to a more pronounced immune suppression, potentially reducing the efficacy of immunotherapies during space missions or in simulated microgravity environments on Earth [66].

These evidences suggest that microgravity conditions can significantly alter immune cell function and responsiveness, which may impact the effectiveness of immunotherapies. Understanding these changes is crucial for developing strategies to mitigate the adverse effects of microgravity on the immune system, especially for astronauts and individuals exposed to similar conditions.

### Effects of microgravity on cytoskeleton and morphology of other cell types

Microgravity has been shown to significantly affect the cytoskeletal structure and morphology of other cell types, including fibroblasts and endothelial cells. In fibroblasts, exposure to simulated microgravity conditions can lead to alterations in the cytoskeleton, extracellular matrix, focal adhesion, and growth factors. For instance, juvenile normal human dermal fibroblasts grown under simulated microgravity exhibit changes in the expression of proteins such as fibronectin, laminin, and collagen, which are crucial for maintaining cell structure and function [67]. These changes are indicative of the fibroblasts’ adaptation to the microgravity environment, which may have implications for tissue engineering and wound healing [67].

Endothelial cells, which are highly sensitive to mechanical forces, also undergo significant changes in microgravity. Studies have shown that microgravity can lead to the depolymerization of actin filaments and microtubules in endothelial cells, affecting their morphology and function [35, 68]. These changes can influence the cells’ ability to regulate vascular homeostasis and respond to inflammatory stimuli. For example, endothelial cells exposed to microgravity show altered expression of adhesion molecules and cytokines, which are critical for maintaining vascular integrity and function [35, 68]. Furthermore, the cytoskeletal remodeling in endothelial cells under microgravity conditions can mimic the effects of mechanical disruption, such as that induced by cytochalasin D [69]. This suggests that the cytoskeleton plays a fundamental role in sensing and responding to gravitational changes, which can have downstream effects on cell signaling and function [69]. Additionally, microgravity-induced changes in endothelial cells can affect their migration and permeability, which are vital for vascular repair and function [70].

Overall, the impact of microgravity on the cytoskeletal structure and morphology of fibroblasts and endothelial cells highlights the importance of understanding these changes for space travel and potential applications in tumor medicine and tissue engineering.

## Impact of microgravity on the biological behavior of cancer cells

Exposure to microgravity leads to significant changes in both the structure and function of cancer cells. Under microgravity conditions, adherent cancer cells detach from the culture surface and aggregate into three-dimensional multicellular spheroids [11, 12, 15]. These spheroids display distinct morphological changes and modified cellular behaviors [43–46]. Unlike normal cells, cancer cells under microgravity exhibit altered differentiation, adhesion, focal adhesion, apoptosis, proliferation, migration, and cell cycle regulation [52, 71, 72]. Microgravity regulates key gene expression pathways in cancer cells, potentially influencing tumorigenesis and offering novel avenues for anticancer strategies [73, 74].

### Hypotheses on the effect of microgravity on tumor cell biological behavior

Although the behavioral changes in tumor cells under microgravity are widely accepted, the underlying mechanisms are not fully understood. Several hypotheses have been proposed to elucidate how microgravity influences the biological behavior of tumor cells.

#### Cytoskeletal reorganization hypothesis

One widely supported hypothesis is that microgravity induces significant changes in the cytoskeleton—a critical structural network that maintains cell shape and mechanical stability [36, 75]. Disruptions in cytoskeletal integrity can disturb cellular mechanical equilibrium, leading to changes in cell morphology, mechanical properties, extracellular matrix (ECM) organization, and intracellular signaling pathways, ultimately resulting in functional alterations. These changes not only disturb normal cell behavior but may also facilitate tumor progression and metastasis [76–78].

#### Gravireceptor-mediated apoptosis hypothesis

Another hypothesis involves the potential presence of a gravireceptor—a proposed sensor within cells that detects mechanical unloading in microgravity. For certain tumor types, such as lung cancer, microgravity has been linked to increased cell death through the activation of apoptosis-regulating genes, including TP53, PTEN, and RB1 [50]. This hypothetical sensor may convert diminished mechanical stimuli into biochemical signals that initiate apoptotic pathways. If identified, such a receptor could serve as a target for therapeutic strategies aiming to trigger selective apoptosis in cancer cells [38, 45, 50].

#### Mitochondrial stress hypothesis

The mitochondrial stress model suggests that microgravity disrupts mitochondrial function, contributing to altered tumor cell behavior. Studies involving astronauts and Earth-bound twins have revealed that prolonged spaceflight is associated with increased oxidative stress and reduced antioxidant capacity, leading to mitochondrial dysfunction [79]. In microgravity, the generation of reactive oxygen species (ROS) is enhanced, which promotes apoptosis [80]. In human promyelocytic leukemia cells, simulated microgravity has been shown to reduce mitochondrial membrane potential, thereby activating the mitochondrial apoptotic pathway [81]. Furthermore, mitochondrial stress may influence cellular metabolism, potentially affecting tumor cell proliferation, survival, and response to therapeutic treatment [82].

### Apoptosis under microgravity

Apoptosis, or programmed cell death, plays a crucial role in processes such as embryonic development, tissue remodeling, and wound repair. It is tightly regulated by the interplay between proteins that either promote or inhibit cell death [83]. In cancer, this regulation is often disrupted, leading to reduced apoptotic activity and enabling tumor cells—including those in malignant gliomas—to persist and evade destruction [84]. As a result, restoring or enhancing apoptotic pathways represents a critical objective in improving cancer therapies [13].

**Fig. 3:**
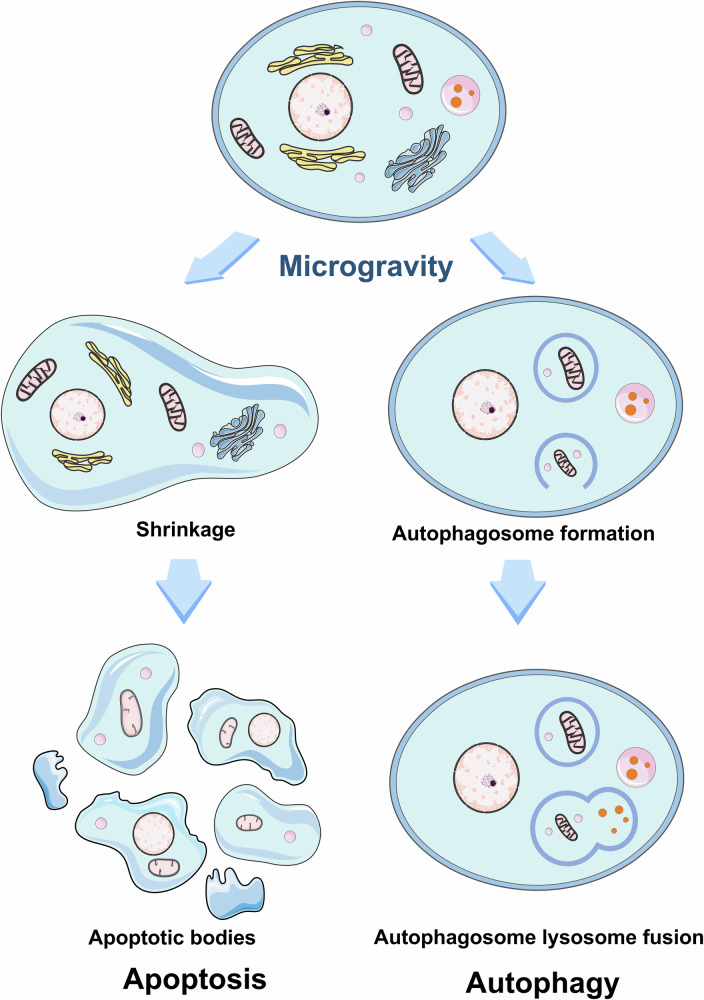
Morphological changes in cancer cells under microgravity-induced apoptosis and autophagy. Microgravity can alter the fate of cancer cells by promoting either apoptosis or autophagy, both of which may contribute to the suppression of tumor growth. During apoptosis, cells undergo shrinkage and fragment into apoptotic bodies, which are efficiently engulfed and removed by macrophages. In contrast, autophagy involves the encapsulation of damaged organelles and proteins within autophagosomes, which subsequently fuse with lysosomes to facilitate degradation. This process supports cellular homeostasis and may act as a barrier to cancer development.

Research has demonstrated that microgravity—both actual and simulated—can significantly influence apoptotic activity [85, 86] (Fig. 3). Numerous studies have reported that microgravity conditions can induce apoptosis in various cancer cell types [50, 54, 87–89]. For instance, simulated microgravity in human lung cancer cells via RPM induces apoptosis within 24 h [50]. Additionally, drug-resistant Jurkat/A4 cells exhibit greater sensitivity to simulated microgravity than wild-type Jurkat cells [90]. Moreover, spheroid cells grown in simulated microgravity show a significantly higher incidence of apoptosis than adherent cells [91]. Furthermore, mesenchymal stem cells (MSCs) exposed to simulated microgravity promoted apoptosis when an anti-cancer vaccine was used in animal models [92]. The pro-apoptotic effects of microgravity appear to be mediated by its influence on key signaling pathways. These effects include the upregulation of apoptosis-associated proteins such as Bax, PARP, and p53, as well as caspase activation. At the same time, microgravity can suppress critical survival pathways, including those regulated by FAK/RhoA-driven mTORC1/NF-κB and ERK1/2 signaling [37, 71, 88]. This dual modulation of cell death and survival mechanisms positions microgravity as a promising experimental model for uncovering novel anti-cancer strategies.

Microgravity can still induce apoptosis in genetically altered cells. In glioma cells (U251MG), microgravity also induces apoptosis by modulating the expression of apoptosis-associated proteins such as p21 and IGFBP-2, which may offer new therapeutic targets for glioma treatment [54]. In addition, a review highlights the potential of microgravity to alter cell proliferation, apoptosis, and gene expression of glioma cells [93]. Similarly, microgravity conditions have been found to trigger DNA damage and mitochondria-mediated apoptosis through ROS generation in human promyelocytic leukemia cells, indicating a potential pathway through which microgravity exerts its effects [81]. Moreover, microgravity has been observed to influence the apoptotic pathways in melanoma cells by suppressing the Uev1A/TICAM/TRAF/NF-κB-regulated anti-apoptosis and p53/PCNA- and ATM/ATR-Chk1/2-controlled DNA-damage response pathways, further supporting its role in promoting apoptosis in cancer cells [37]. Overall, microgravity disrupts tumor cell proliferation and promotes programmed cell death, suggesting its potential in developing novel cancer therapies. For detailed effects across tumor types, refer to Table 1.

**Table 1:** Effects of microgravity on apoptosis and autophagy in human cancer cell lines.

Tumor / Cell line	G-condition	Culture time	Apoptosis/autophagy-related results	Possible mechanism	Ref.
Lung cancer					
*H460*	RPM	48 h	Increased apoptosis, stemness loss, multicellular sphere formation	-	[113, 114]
*CRL-5889*	RPM	72 h	Increased apoptosis	-	[50]
Colorectal cancer					
*DLD-1*	RCCS	48 h	Increased apoptosis, autophagy activation, PTEN ↑ , FOXO3 ↑ , AKT↓	PTEN/FOXO3/AKT pathway	[15]
*HCT-116*	RCCS	72 h	Increased autophagy, PTEN ↑ , FOXO3 ↑ , ATG5/7/12/16L1 ↑ , YAP ↑ , OCT4A ↑ , SOX2 ↑ , Nanog ↑ , NKx-2.5↑	Hippo pathway	[98]
Gastric cancer					
*SGC-7901*	CN	72 h	Increased autophagy	-	[115]
Thyroid cancer					
*ML-1*	RPM	24 h	P53 ↑ , Fas ↑ , Bax ↑ , Bcl-2↓	-	[116]
*FTC-133*	RPM	72 h	No apoptosis detected	-	[117]
*ONCO-DG1*	CN	24 h	Bax ↑ , Bcl-2 ↓ , caspase-3 activation, destruction, and redistribution of mitochondria	Fas-dependent apoptotic pathway, extrinsic and intrinsic apoptotic pathways	[118]
Breast cancer					
*MCF-7*	RPM	24 h	p53 ↑ , Fas ↑ , Bax ↑ , caspase-8↑	HMOX-1 and NFκB	[48]
*MDA-MB-231*	RPM	72 h	Bax ↑ , Bcl-2↓	-	[119]
	RCCS	7 d	Bcl-2 ↓ , MMP9 ↓ , cyclin D3↑	-	[120]
Hepatoblastoma					
*HepG2*	3D-CN	72 h	Bax ↓ , CDKN1A ↓ , PTEN ↑ , DRAM1 ↑ , PRKAA1 ↑ , autophagy activation	p53-independent mechanisms	[99]
Glioma					
*U251*	CN	72 h	p21 ↑ , IGFBP-2↓	-	[54]
	CN	24 h	Bcl-2 ↓ , Bnip3 ↓ , cleaved caspases 3/9↑	FAK/RhoA/Rock and FAK/Nek2	[11]
*C6*	RPM	1-24 h	Translocation of Bax and Bcl-2	-	[121]
Melanoma					
*BL6-10*	RPM	24 h	Bcl-2 ↓ , Bnip3 ↓ , caspases 3/7/8↑ Increased apoptosis	NF-kB and ATM/ATR-Chk1/2 pathways	[37]
Promyelocytic leukemia					
*HL-60*	RCCS	72 h	cleaved-caspase-3 ↑ , Bax/Bcl-2 ratio ↑ , ROS ↑ , dissipation of mitochondrial membrane, DNA damage	ATM/ATR-Chk1/2 and Ku70/80 and DNA-PK-mediated apoptosis ↑	[81]
	RWV	48 h	Increased apoptosis	VEGFR-2/VEGF-A pathway	[122]
Hodgkin’s lymphoma					
*L-540, HDLM-2*	RWV	48 h	Increased autophagy, ROS ↑ , NADPH oxidase family gene ↑ , mitochondrial mass↓	AMPK/Akt/mTOR and MAPK pathway	[100]
Seminoma					
*TCam-2*	RPM,	24/48 h	Autophagy activation	-	[101]

↑ upregulation, ↓ downregulation, *3D* three dimensional, *CN* clinostat, *RCCS* rotating cell culturing system, *RPM* random positioning machine, *RWV* rotating wall vessel.

### Microgravity-induced autophagy

A key obstacle in conventional cancer treatments is the ability of tumor cells to evade apoptosis [94]. Autophagy, a distinct form of programmed cell death, may offer alternative therapeutic strategies for cancer treatment [78, 94]. Studies suggest that microgravity can upregulate autophagy across various cell types, contributing to muscle atrophy, ocular pathogenesis, and other spaceflight-associated diseases [95–97]. Interestingly, targeting autophagy in cancer cells under microgravity could inspire new strategies for combating cancer.

Table 1 summarizes the effects of microgravity on apoptosis and autophagy in different human cancer cell lines. In human colorectal cancer cells (HCT116) cultured using a RCCS to simulate microgravity, autophagy was enhanced, as evidenced by increased ATG expression [98]. Similarly, hepatoblastoma cell lines subjected to simulated microgravity exhibited upregulation of LC3-II relative to LC3-I and a downregulation of mTOR expression, indicating autophagy activation [99]. Notably, these changes were also seen in hepatoblastoma cells expressing mutant p53, a key regulator of both apoptosis and autophagy [99]. Jeong et al. reported that Hodgkin’s lymphoma cells exposed to two days of simulated microgravity showed autophagy induction via the AMPK/Akt/mTOR and MAPK signaling cascades [100]. Furthermore, colorectal cancer cells formed spheroids after 6–8 hours of exposure to simulated microgravity in RCCS [15]. When returned to normal gravity, these spheroids displayed deregulated autophagy [15]. In seminoma TCam-2 cells, microgravity conditions promoted substantial autophagy activity and disrupted the organization of actin microfilaments [101].

**Fig. 4:**
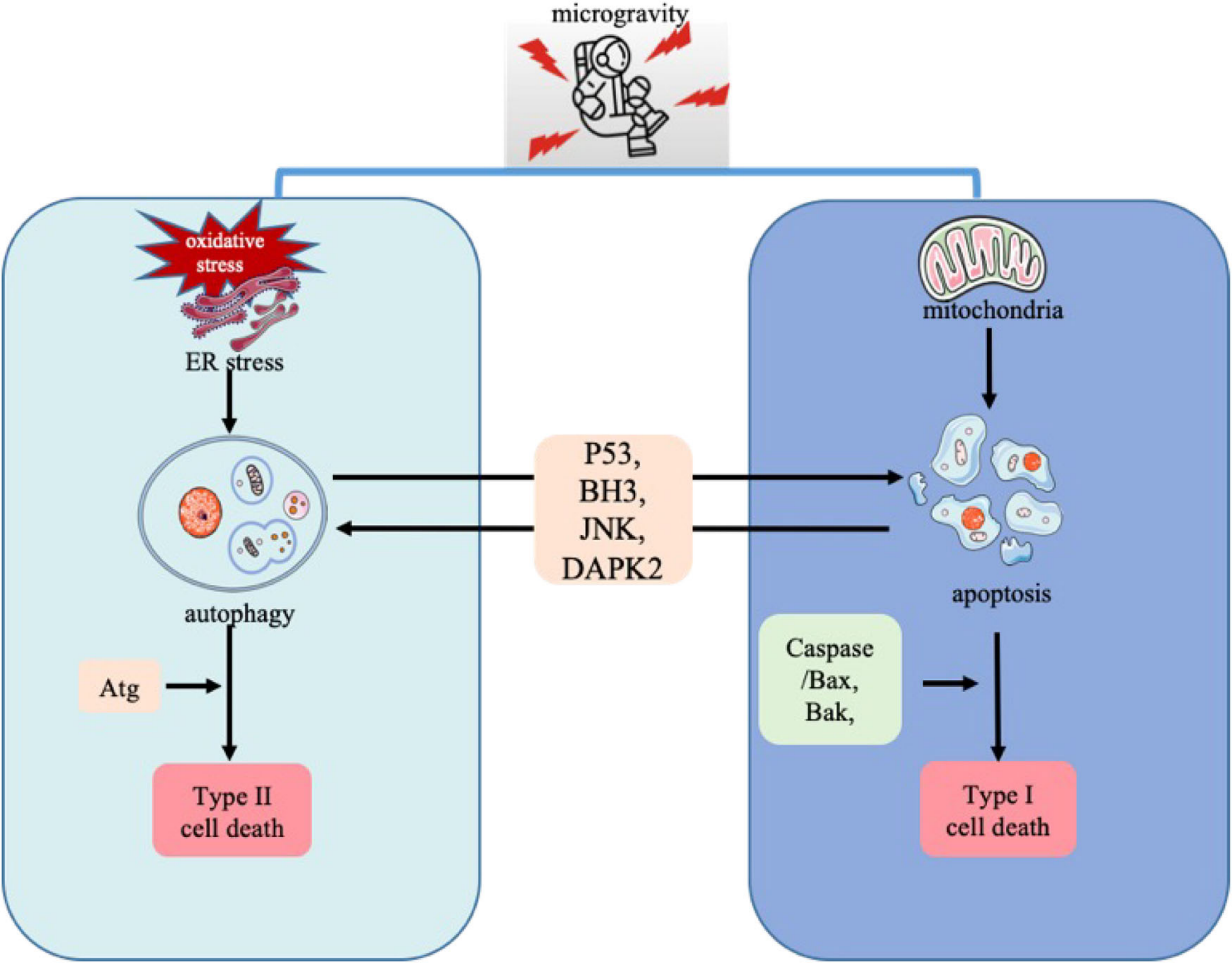
Potential interconnection between apoptosis and autophagy under microgravity. Microgravity can trigger cellular stress responses such as oxidative stress and endoplasmic reticulum stress, which may lead to the activation of autophagy, potentially mediated by ATG [15, 98, 123]. Additionally, microgravity influences mitochondrial function, particularly by increasing the permeability of the mitochondrial outer membrane. This can cause the release of cytochrome c, a critical event in the intrinsic apoptotic pathway, which is regulated by key proteins such as caspases, Bax, and Bak [11, 48, 81, 99, 118]. Under standard physiological conditions, apoptosis and autophagy generally act in opposition to maintain cellular balance. However, in microgravity, their relationship appears more complex [102]. Crosstalk between these pathways may be facilitated by mechanisms such as proteolytic cleavage of the transcription factor p53 or through interactions between BCL2 family proteins and the BH3 domain [102]. These processes may also be influenced by signaling pathways involving kinases like JNK and DAPK2 [102].

Given this evidence, it is essential to investigate the triggers of tumor cell death under microgravity, particularly the interconnected roles of apoptosis and autophagy. Under physiological conditions, the crosstalk between autophagy and apoptosis may be mediated through proteolytic cleavage of the transcription factor p53 or through interactions involving BCL2 family proteins at the BH3 domain, mechanisms possibly regulated by kinases such as JNK and DAPK2 [102]. Further research is needed to clarify how autophagy and apoptosis are interlinked under microgravity and to explore the molecular mechanisms at play. The structural features of apoptosis and autophagy observed in tumor cells exposed to microgravity are depicted in Fig. 3, while Fig. 4 illustrates their potential relationship under microgravity.

### Impact of microgravity on the cell cycle

Microgravity has been found to significantly affect the cell cycle in cancer cells, primarily by impairing mitochondrial function, disrupting normal progression, and suppressing cellular proliferation [103]. Tumor cells cultured under simulated microgravity show distinct changes in the expression of proteins that regulate the cell cycle [71]. For instance, exposure to microgravity results in the downregulation of cyclin D1 and cyclin B1 in breast and colorectal cancer cells—two key regulators of cell cycle checkpoints [71]. In BL6-10 melanoma cells, reduced expression of ATR, ATM, and CDK1/2 during the transition from the S-phase to the G2-phase has been observed under microgravity conditions, which corresponds with a notable decline in the population of G2-phase cells [37]. Additionally, the PCNA-CDK-cyclin complex, a crucial component required for seamless progression through the cell cycle, is diminished in microgravity-exposed cells [104]. This downregulation is accompanied by increased levels of the P21 protein, a known inhibitor of DNA synthesis and cell cycle progression [104]. P21 can bind to PCNA, thereby inhibiting CDK function and arresting the cell cycle in the G1 phase [104]. Microgravity has also been shown to reduce the colony-forming ability of melanoma, colorectal, and leukemia cancer cell lines [37, 71]. These findings suggest that microgravity may hinder tumor cell proliferation and colony formation by modulating the expression of cell cycle-related genes and proteins.

### Tumor cell adhesion in microgravity

Under normal gravity, epithelial-mesenchymal transition involves the loss of intercellular adhesion and enhanced interactions with the extracellular matrix (ECM) [105]. The ECM, adhesion molecules, and cytoskeleton components constitute a highly responsive network that adjusts to gravitational changes by modulating key signaling pathways [71]. Notably, changes in adhesion-related gene expression are among the earliest cellular responses to microgravity, preceding the onset of apoptosis. In real microgravity, human breast cancer cells exhibit rapid remodeling of adhesion and cytoskeletal proteins within six minutes, marked by a decline in E-cadherin levels, a molecule often associated with reduced metastatic potential [77].

Similarly, under simulated microgravity, both intercellular and focal adhesions are disrupted [106]. This is accompanied by inhibition of signaling pathways such as FAK, RhoA, and mTORC1, along with activation of the AMPK pathway, collectively contributing to decreased melanoma cell proliferation and metastatic behavior [107]. The suppression of cell adhesion under microgravity is further evidenced by reduced expression of adhesion markers like E-cadherin and integrin-β1 [106]. Proteomic studies in MCF-7 breast cancer cells have revealed downregulation of E-cadherin, claudin-3, and integrin-β4, along with reduced levels of their negative regulators, indicating that E-cadherin may be a key regulator of multicellular spheroid formation in microgravity [108]. These cellular adaptations likely represent responses to the abnormal mechanical environment of microgravity, in which altered external forces are translated into internal signals that affect cell morphology, adhesion properties, and cytoskeletal structure [106, 108]. The deregulation of adhesion-related proteins appears to play a central role in orchestrating these phenotypic changes [106].

### Metabolic rewiring under microgravity

In human hepatic and biliary tree stem/progenitor cells, Costantini et al. demonstrated that the RCCS significantly enhances glycolytic metabolism [109]. Under RCCS-simulated microgravity conditions, metabolomic profiling of HGC-27 gastric cancer cells revealed paradoxical metabolic reprogramming. RCCS exposure promoted glycolytic flux while suppressing oxidative phosphorylation, suggesting potential inhibition of the Warburg effect in this specific tumor model [110]. Proteomic analysis of murine microgravity models further identified hyperactivation of rate-limiting glycolytic enzymes, including hexokinase and phosphofructokinase, driving enhanced glucose metabolism under SMG conditions [111]. However, the precise impact of SMG on tumor metabolism—particularly its regulatory effects on the Warburg effect—requires further validation through systematic investigations integrating multi-omics approaches and functional metabolic assays.

## The future prospects and current dilemma of microgravity research

Microgravity presents exciting potential for future clinical applications, especially in areas such as wound repair, tissue regeneration, pharmaceutical development, and cancer therapy. As a novel experimental approach, it has produced encouraging early results, prompting increased interest in its role in regenerative medicine and innovative oncological treatments [13, 72, 112]. Despite these advancements, the practical use of microgravity in clinical settings remains in the early stages. Simulating microgravity requires sophisticated and costly equipment, and there is a scarcity of long-term studies to support its widespread application. Future progress in space technologies and biomedical research is expected to improve experimental systems and broaden their accessibility. To bridge the gap between laboratory research and clinical implementation, comprehensive preclinical studies—particularly those conducted aboard space platforms—will be critical. Establishing robust and scalable microgravity-based methodologies will be an important direction for translating this promising technology into healthcare solutions.

## Conclusions

Microgravity provides a unique three-dimensional in vitro model that closely mimics the tumor microenvironment. Studies under microgravity have revealed critical insights into cancer cell behavior, including apoptosis, autophagy, adhesion, and cell cycle regulation. These findings challenge traditional research paradigms and suggest that microgravity could be instrumental in developing novel cancer therapies and prevention strategies. As research progresses, microgravity may become a vital tool in advancing cancer treatment and improving patient outcomes.
